# Evaluation of Gene Expression of Mirna 21 and 98 Effect on Liver Functions in Fibrotic Patients: Evaluation of Gene Expression of Some miRNAs and Liver Functions in Fibrotic Patients

**DOI:** 10.31661/gmj.v14i.3833

**Published:** 2025-03-17

**Authors:** Baraa Jalil Saeed, Wejdan Thamer Mahdi, Mohammed Abdulwahab Al-Askeri

**Affiliations:** ^1^ Department of Biology, University of Al-Qadisiyha, Al Diwaniyah, Iraq

**Keywords:** Gene Expression, Mirna 21, Mirna 98, Liver Fibrosis, RT- PCR

## Abstract

**Background:**

Liver fibrosis is one of concerning outcomes of hepatitis with a complex interaction of different pathological processes including the epigenetics. MicroRNAs (miRNA) like miRNA 21 and 98 are recently being emphasized in era of epigenetics of liver diseases. The current study aims to evaluate liver function and the role of miRNA 21 and 98 in the occurrence of liver fibrosis.

**Materials and Methods:**

This was a case-control study comparing liver fibrosis patients with healthy adults with intact liver function as the control group. Laboratory and clinical tests were performed to evaluate liver function, and the diagnosis of viral hepatitis was confirmed using ELISA kits. Reverse transcription polymerase chain reaction (RT-PCR) was used to analyze the expression levels of microRNA 21 and 98, following the extraction of RNA from the samples.

**Results:**

Our study included 44 cases and 44 controls. Results revealed significant differences in demographic, clinical, and biochemical properties. Cases had higher mean BMI (34.36 ± 9.14 kg/m2, P=0.0486), higher levels of TSB, ALP, ALT, AST, and CRP, and lower albumin levels (P 0.05 for all). Gene expression analysis showed higher miRNA 21 (1.14, P 0.05) and lower miRNA 98 (1.68, P 0.05) in cases compared to controls. Among case group, females had higher expression of miRNA 21 (1.082 ± 0.23) and miRNA 98 (1.895 ± 0.41) compared to males (P0.05). Additionally, cases infected with HBV had higher expression of miRNA 21 (1.201 ± 0.31) and miRNA 98 (2.985 ± 0.56) compared to those infected with HCV (P0.05).

**Conclusion:**

In conclusion, miRNA 21 and miRNA 98 might be associated with liver fibrosis; while the exact pathophysiological explanation reminds unclear.

## Introduction

The term "Liver disease, or hepatic disease" applies to many illnesses and disorders that cause the liver to either function improperly or stop functioning completely [1]. When diagnosing or tracking the progression of liver disease or damage, a blood test known as a liver function test may be helpful. The tests measure the blood levels of certain enzymes or proteins [2]. Liver fibrosis is characterized by the accumulation of extracellular matrix proteins, which can lead to the formation of scar tissue and ultimately, cirrhosis [3]. The pathogenesis of liver fibrosis involves a range of cellular and molecular mechanisms, including the activation of hepatic stellate cells [3]. Additionally, insulin resistance and non-alcoholic fatty liver disease have been identified as significant risk factors for the development of liver fibrosis [1][2][3].

Many miRNAs are associated with systemic and organ-specific fibrosis in the liver. The expression of individual miRNA in plasma or serum is helpful for detecting liver fibrosis [4][5]. MiR-98 (microRNA 98) is an RNA gene affiliated with the miRNA class. MiR-98 belongs to the let-7 miRNA family, which was first discovered to control the developmental timing of cell proliferation and differentiation in Celegans. MiR-98 are endogenous noncoding RNAs of about 22 nucleotides that bind to complementary sequences found in the 3-prime UTRs of target mRNAs, resulting in mRNA cleavage or translational suppression. Recently, altered expression of miR-98 has been found in several carcinomas [5][6].

The MIR21 gene encodes microRNA 21, also called has-miRNA21 or miR-21, which is a microRNA found in mammals. The human microRNA-21 gene is located on the plus strand of chromosome 17q23.2 (55273409-55273480), inside a gene producing TMEM49 (also named vacuole membrane protein) [7][8].

Its genetic targets have linked it to various illnesses, including neoplastic and non-neoplastic diseases. Tropomyosin 1, Phosphatase and Tensin Homolog (PTEN), and Programmed Cell Death 4 (PDCD4) are three of miR-21's primary targets [9][10]. Studies have documented the increased expression of miRNAs, such as miR-21, in hepatitis and liver cancer. MiR-21's ability to induce fibrogenesis affects muscles and other organs, including the heart, lungs, kidneys, and liver [10][11]. As the current understanding of liver fibrosis is hindered by the complexity of its pathological processes, including the involvement of epigenetics, and despite the recent emphasis on miRNAs like miRNA 21 and 98 in the era of epigenetics of liver diseases, there is still a significant gap in knowledge regarding the specific role of these miRNAs in the occurrence of liver fibrosis; We attempted to identify the potential miRNA 21 and 98 associated with hepatic fibrosis using RT-PCR-dependent gene expression, in addition to evaluating the liver function of fibrotic patients in our population.

## Material and Methods

**Table:** Table1. RT--PCR Primers with Their Sequence

**Primer**		Sequence (5’-3’)
**miRNA universal RT primers**		GTCGTATCCAGTGCAGGGTCCGAGGT ATTCGCACTGGATACGACTTTTTTTTTTTVN
**miR-98** **qPCR primer**	F	AACCTCCTGAGGTAGTAAGTTGTAT
	R	GTCGTATCCAGTGCAGGGT
**miR-21** **qPCR primer**	F	AACAAGAGTTCTTCAGTGGCAAG
	R	GTCGTATCCAGTGCAGGGT
**GAPDH** **qPCR primer**	F	TCACCAGGGCTGCTTTTAAC
	R	TGACGGTGCCATGGAATTTG

**Table:** Table2. Comparison of Demographic, Clinical, and Biochemical Properties between Cases and Healthy Controls

**Properties**	**Cases** **N=44**	**Control** **N=44**	**P-value**
**Age range**	29 - 78	25 - 76	-
**Mean Age ± SD**	61.16 ± 11.16	55.6 ± 11.14	0.226
**Gender (Males)**	30 (68%)	26 (59%)	0.077
**Gender (Females)**	14 (32%)	18 (41%)	0.077
**BMI (Kg/M2) (Mean ± SD)**	34.36 ± 9.14	23.87 ± 2.87	0.0486
**Hepatitis**	7 (16%)	-	-
**Chronic diseases**	40 (91%)	-	-
**Smoking**	27 (61%)	17 (39%)	0.038
**TSB (mg/dl) (Mean ± SD)**	15.3 ± 2.50	0.488 ± 0.09	0.031
**ALP (U/L) (Mean ± SD)**	370 ± 108.59	122.56 ± 10.96	0.022
**ALT (U/L) (Mean ± SD)**	42.36 ± 5.97	26.36 ± 4.95	0.037
**AST (U/L) (Mean ± SD)**	47.76 ± 2.96	11.92 ± 2.16	0.019
**Albumin (g/dl) (Mean ± SD)**	1.78 ± 0.42	3.90 ± 0.44	0.0219
**CRP (mg/L) (Mean ± SD)**	15.45 ± 2	6.76 ± 0.828	0.043

### Study Design and Settings

This study employed a case-control design to investigate the relationship between liver fibrosis and molecular biomarkers. The study was conducted at Al-Diwaniyah Teaching Hospital, outpatient clinics, Medical City in Baghdad, and the Gastrointestinal Unit (GIT) at Al-Hakim Hospital/Najaf Al-Ashraf, from September 3, 2022, to October 15, 2023.

### Eligibility Criteria

The study included 44 cases with liver fibrosis and 44 healthy controls. The cases were selected based on clinical diagnosis, and the controls were chosen from the same population to match the cases in terms of age and sex. The study used a 1:1 matching ratio, where each case was matched with a control of the same age and sex. The eligibility criteria for cases included a confirmed diagnosis of liver fibrosis, while the controls had no history of liver disease.

### Outcomes, Exposures, Predictors, Potential Confounders, and Effect Modifiers

The primary outcome was the expression of miR-98 and miR-21 in liver fibrosis patients compared to healthy controls. The exposure of interest was liver fibrosis, and the potential confounders included age, sex, and hepatitis status. The diagnostic criteria for liver fibrosis were based on clinical and laboratory findings.

### Data Sources/Measurement

The data sources included blood samples collected from participants, which were used for liver function tests, hepatitis tests, C-reactive protein tests, and molecular studies. The methods of assessment included immunoassays, chromatography, and quantitative real-time PCR (qRT-PCR). The assessment methods were comparable between cases and controls.

### Molecular Study

RNA was extracted from blood samples using the TRIzol® reagent kit, according to the manufacturer's instructions. The extracted genomic RNA was checked using a Nanodrop Spectrophotometer, which verifies RNA concentration and estimates RNA purity by measuring absorbance at 260 nm and 280 nm wavelengths.

The extracted RNA was processed with DNase I enzyme to eliminate any small amounts of genomic DNA from the eluted total RNA. Following the method laid out by the Promega corporation in the United States of America, this procedure was executed utilizing samples and a DNase I enzyme kit. The reverse transcription step was then initiated, where the purified RNA was converted into complementary DNA using a reverse transcriptase enzyme, specifically the GoScript Reverse Transcriptase kit, in a 20 μL reaction mixture containing 1 μg of RNA, 1 μL of oligo(dT) primer, and 1 μL of reverse transcriptase, which was incubated at 42°C for 60 minutes to synthesize the first-strand cDNA. The qPCR primers for miR-98 (MIMAT0000096) and miR-21 (MIMAT0004495) were designed in this study using (the Sanger Center miRNA Database Registry) to select the miRNA sequence and the miRNA primer design tool. In this study, gene qPCR Housekeeping (GAPDH) (NM_001256799.3) was designed using NCBI-Database and Primer3 plus design online.

According to Table-1, these primers were supplied by the Macrogen company in Korea, which used the GoTaq® qPCR Master mix kit to make the qPCR master mix. Then, before being put in the Mini optic on the Real-Time PCR system, Using an ExiSpin vortex centrifuge, the components of the qPCR master mix were put into tubes for qPCR strip plate and mixed for three minutes. After that, the qPCR plate was loaded and subjected to the following mocycler protocol for miRNA genes or GAPDH genes within the following: the initial denaturation TM step at 95°C for 5 minutes (1 cycle), denaturation step2 at 95 °C for 20 seconds (45 cycles), annealing\extension/ detection (scanning) at 60 °C for 30 seconds (45 cycles). Relative quantification gene expression levels (the ΔCT technique using a reference gene) were utilized to analyze the qRT-PCR data for the target and housekeeping genes, as mentioned by Livak and Schmittgen [12]. Like the following equation:

∆CT (Test)=CT (target gene, test)- CT (the HKG gene, test)

∆CT (Control)=CT (target gene, control) - CT (HKG gene, control)

∆∆CT=∆CT (Test)- ∆CT (Control)

Fold change (target / HKG)=2-CT ∆∆CT

### Statistical Analysis

The present research included SPSS IBM SPSS Statistics for Windows, version 19 (IBM Corp., Armonk, N.Y., USA), to analyze data. Qualitative data was compared by chi-square; while quantitative ones were compared by independent t test. Statistically significant associations were defined as differences lower than 0.05.

### Ethical Approval

Approval was formally obtained from the hospital before starting work, and verbal consent was obtained from patients before collecting samples.

## Result

**Table:** Table3. Comparison Mean of Gene Expression of miRNA 21, 98 between Cases and Control

**Gene expression**	**Case-control**	**Mean CT(gene)**	**Mean CT(gapdh)**	**Mean ∆CT** **(test)**	**Mean ∆CT(control)**	**Mean Fold change (2^-∆∆CT^) **
**miRNA 21**	**Cases**	29.6	27.2	-2.38	2.38	1.14
	**Control**	27.6	27.8	-0.145	2.38	0.33
**miRNA 98**	**Cases**	26.16	27.23	-1.08	-1.08	1.68
	**Control**	28.95	27.78	1.17	-1.08	6.30

significant association in compared with controls (P<0.05)

**Table:** Table4. Gene Expression of miRNA 21 and miRNA 98 in Case Group

**Category**	**miRNA 21 (Mean ± SD)**	**P-value**	**miRNA 98 (Mean ± SD)**	**P-value**
**Female**	**1.082 ± 0.23**	**0.041**	**1.895 ± 0.41**	**0.011**
**Male**	**0.921 ± 0.17**		**1.234 ± 0.29**	
**HBV**	**1.201 ± 0.31**	**0.008**	**2.985 ± 0.56**	**<0.001**
**HCV**	**0.883 ± 0.21**		**0.310 ± 0.15**	

**Figure-1:**
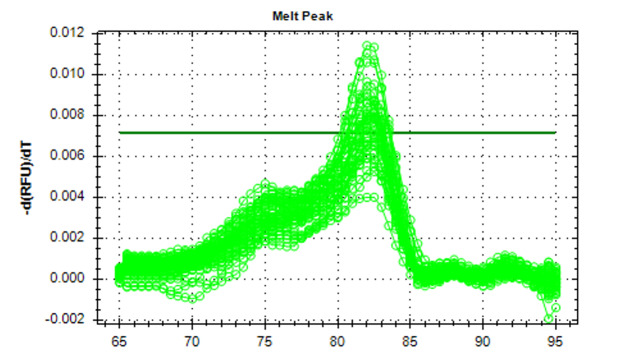
Figure 1. The real-time PCR melting plot of miRNA 21 gene expression that showed the qPCR specificity at (82°C)

**Figure-2:**
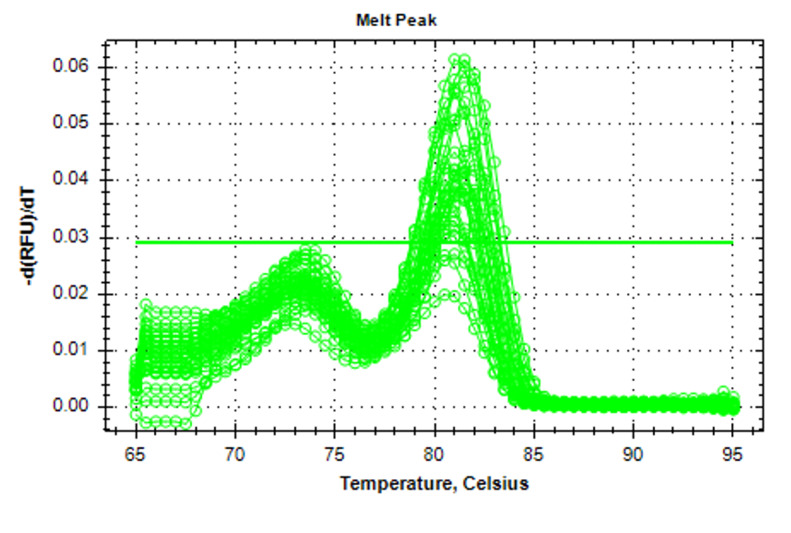
Figure 2. The real-time PCR melting plot of miRNA 98 gene expression that showed the qPCR specificity at (81°C)

The current research is a case-control study, that involved 44 patients suffering from liver fibrosis. As shown in Table-2, a comparison between cases and healthy controls revealed significant differences in various demographic and clinical properties. The mean age of cases was 61.16 ± 11.16 years, with a range of 29-78 years, whereas controls had a mean age of 55.6 ± 11.14 years, with a range of 25-76 years (P=0.226). The gender distribution showed that 68% of cases were males, compared to 59% in controls (P=0.077).

Additionally, cases had a higher mean BMI (34.36 ± 9.14 kg/m2) compared to controls (23.87 ± 2.87 kg/m2) (P=0.0486). Clinical assessments indicated that 16% of cases had hepatitis (P=0.017), 91% had chronic diseases (P=0.004), and 61% were smokers (P=0.038). Biochemical markers also showed significant differences, with cases having higher levels of TSB, ALP, ALT, AST, and CRP, and lower levels of albumin, compared to controls (P<0.05 for all). These findings suggest significant differences between cases and healthy controls in terms of demographic, clinical, and biochemical properties.

The results from the gene expression analysis (Figures-1 and -2) showed increased mean fold change (2-∆∆CT) for miRNA 21 in patients (1.14) compared to controls (0.33). At the same time, we found a lower mean fold change for miRNA 98 in patients (1.68) compared with healthy people, (6.30) which led to the emergence of significant differences (P<0.05) as in Table-3.

As shown in Table-4, the gene expression of miRNA 21 and miRNA 98 in the case group revealed significant differences based on sex and type of viral infection. Female cases had higher expression of miRNA 21 (1.082 ± 0.23) and miRNA 98 (1.895 ± 0.41) compared to males, with P values of 0.041 and 0.011, respectively. Additionally, cases infected with HBV had higher expression of miRNA 21 (1.201 ± 0.31) and miRNA 98 (2.985 ± 0.56) compared to those infected with HCV, with P values of 0.008 and <0.001, respectively.

## Discussion

The present research found that the gene expression of miR-21 was higher in persons with liver fibrosis than healthy individuals. Nevertheless, there is variation and diversity in the expression of miR-21 in different kinds of liver disease, depending on the research. Several biological processes, including inflammation, fibrosis, and cancer, involve an upregulation of MiR-21. The significance of miR-21 in many liver illnesses is becoming increasingly shown by increasing evidence [13]. MiR-21 dysregulation is prevalent in several kinds of chronic hepatic diseases.

When considering miR-21 as a biomarker for certain liver illnesses or a treatment target, there are several possible problems to consider [14][15].

Prior studies have shown that microRNAs produced by the host may regulate viral infections positively or negatively by targeting viral genomes or cellular components [16]. According to the current results, miRNA 21 and 98 levels were higher in those with HBV infection and lower in those with HCV infection.

Viral infections may affect the expression levels of cellular miRNA and provide a conducive environment for their survival and pathogenic effects. Studies have shown that levels of miR-21 in the blood serum of individuals infected with the Hepatitis B virus (HBV) were elevated [17][18]. Though there was no conclusive evidence linking miR-21 to HBV infection or replication, certain studies indicated that miR-21 played a crucial role in the non-tumor to-tumor transformation induced by the HBV x protein (HBx). The transformation occurred through the PTEN/PI3K/Akt signaling pathway [19]. Our outcomes are similar to those of an earlier study that found that liver cells lines and fundamental humans liver cells exhibit an increase miR-21 when they are exposed to HCV [20]. Findings from a clinical study showed miR-21 expression in liver tissues was higher in increased viral loads and levels of fibrosis in HCV patients' liver biopsies [21].

The study by Chen et al. demonstrated that miR-21 has a role in negatively regulating the signaling of IFN-α during HCV infection by inhibiting the activity of myeloid differentiation primary response 88 (MyD88) and Interleukin1 Receptor Associated Kinase 1 (IRAK1) [22].

In the current study, gene expression of miRNA 98 was lower in fibrotic patients, especially males. In the same line, activated hepatic stellate cells (HSCs) and multiple hepatic fibrotic models demonstrated reduced miR-98 expression, according to research by Wang et al. [23]. In addition, the administration of miR-98 agomir reduced hepatic fibrosis and suppressed the expression of HLF in mice. Their research revealed that miR-98 protects liver fibrosis by specifically targeting HLF and controlling a novel signaling pathway, including HIF-1α, TGF-β, and Smad2/3 [23]. A possible predictor of liver fibrosis development was identified by the research conducted by Ma et al. [24] as miR-98-5p. Overexpression of miR-98-5p inhibited the activation of HSCs, whereas activated LX2 cells showed a decrease in miR-98. By interacting with TGFbR1 and blocking the TGFb1/Smad3 signaling pathway, MiR-98-5p inhibits liver fibrosis. In addition, 70 patients with chronic HBV infection and 29 healthy persons were measured in the study to test blood levels of miR-98. People with liver fibrosis had a much lower miR-98 in their serum than healthy controls and HBV carriers ]25[.

There is some evidence that miR-98-5p promotes cell apoptosis and inhibits hepatoma cell growth, at least in part, by inhibiting its target gene IGF2BP1]25[. According to another research, miR-98 inhibits glioma cell motility and invasion. It was also shown that miR-98 targets the IκB kinase IKKε in glioma cells ]26[. Nevertheless, there is a scarcity of studies regarding the role of miR-98-5p in the process of liver fibrosis. By targeting activating receptor-like kinase-4 and matrix metalloproteinase-11, siragam and colleagues discovered that miR-98 decreased breast cancer cells proliferation, growth, survival, invasion, and angiogenesis ]27[. Yang et al. found that miR-98 expression is down-regulated in non-small cell lung cancer, and P21-activated protein kinase 1 (PAK1) expression is significantly elevated [28].

Our study found that the mean fold change (2-∆∆CT) of genes miRNA 21 and miRNA 98 increased in females. This result confirms that the genetic expression of these genes is directly or indirectly linked to sex hormones, and in general we did not find sufficient studies to explain this. Szabo and Bala, in their study on miRNAs, indicated that the patient’s gender was associated with the gene expression of miRNAs [29].

## Conclusion

The findings of the present investigation indicate that chronic diseases associated with obesity and smoking are the main causes of liver fibrosis. Genetically, miRNAs 21 and 98 have been linked to liver fibrosis, but in an opposite role, since an increase in the gene expression of miRNA 21 or a decrease in miRNA 98 coincides with the appearance of fibrosis, particularly in individuals infected by HBV. We also found a clear disturbance in liver function due to the high level of liver enzymes and CRP in patients compared to healthy people.

## Conflict of Interest

No conflict of interest exists.
